# Multi-Armed Star-Shaped Block Copolymers of Poly(ethylene glycol)-Poly(furfuryl glycidol) as Long Circulating Nanocarriers

**DOI:** 10.3390/polym15122626

**Published:** 2023-06-09

**Authors:** Yasuhiro Nakagawa, Kotaro Ushidome, Keita Masuda, Kazunori Igarashi, Yu Matsumoto, Tatsuya Yamasoba, Yasutaka Anraku, Madoka Takai, Horacio Cabral

**Affiliations:** 1 Graduate School of Engineering, The University of Tokyo, 7-3-1 Hongo, Bunkyo-ku, Tokyo 113-8656, Japan; nakagawa.y.ay@m.titech.ac.jp (Y.N.); ushidome-kotaro081@g.ecc.u-tokyo.ac.jp (K.U.); masudakeita@g.ecc.u-tokyo.ac.jp (K.M.); anraku@bmw.t.u-tokyo.ac.jp (Y.A.); takai@bis.t.u-tokyo.ac.jp (M.T.); 2 School of Materials and Chemical Technology, Tokyo Institute of Technology, 2-12-1 Ookayama, Meguro-ku, Tokyo 152-8550, Japan; 3 Department of Otorhinolaryngology and Head and Neck Surgery, Graduate School of Medicine and Faculty of Medicine, The University of Tokyo, 7-3-1 Hongo, Bunkyo-ku, Tokyo 113-8655, Japan; tempesta50.1@gmail.com (K.I.); yumatsumoto@mac.com (Y.M.); tyamasoba-tky@umin.ac.jp (T.Y.)

**Keywords:** nanomedicine, star polymers, Diels–Alder, poly(ethylene glycol), pharmacokinetics

## Abstract

Multi-arm star-shaped block copolymers with precisely tuned nano-architectures are promising candidates for drug delivery. Herein, we developed 4- and 6-arm star-shaped block copolymers consisting of poly(furfuryl glycidol) (PFG) as the core-forming segments and biocompatible poly(ethylene glycol) (PEG) as the shell-forming blocks. The polymerization degree of each block was controlled by adjusting the feeding ratio of a furfuryl glycidyl ether and ethylene oxide. The size of the series of block copolymers was found to be less than 10 nm in DMF. In water, the polymers showed sizes larger than 20 nm, which can be related to the association of the polymers. The star-shaped block copolymers effectively loaded maleimide-bearing model drugs in their core-forming segment with the Diels–Alder reaction. These drugs were rapidly released upon heating via a retro Diels–Alder step. When the star-shaped block copolymers were injected intravenously in mice, they showed prolonged blood circulation, with more than 80% of the injected dose remaining in the bloodstream at 6 h after intravenous injection. These results indicate the potential of the star-shaped PFG-PEG block copolymers as long-circulating nanocarriers.

## 1. Introduction

To achieve successful therapeutic effects, a sufficient amount of the active drug must reach the biological target, and the effective dose must be maintained for the duration of the treatment. Such a process presents several demanding barriers for conventional drugs, including accelerated degradation in in vivo environments, renal excretion, immune elimination, and extensive off-target biodistribution following systemic administration [1]. Thus, several types of nanocarriers have been developed for improving the stability of drugs in biological environments and enhancing the delivery to cells and tissues by modulating the pharmacokinetics and drug activation [2,3,4,5,6]. Such nanocarriers can control the in vivo performance by adjusting their physicochemical–geometrical characteristics, including the size, surface chemistry, installation of ligands, and introduction of stimuli-responsive chemistry [7,8,9].

Polymer-based nanocarriers have shown high capability for protecting drugs and selectively delivering them to target tissues [10,11,12,13,14,15]. Among polymeric structures, multi-armed star polymers, i.e., polymer nano-architectures with linear arms radially branched from a center with multiple initiating points [16], are receiving much attention for efficient drug delivery by the optimization of the length, number, composition, and primary structures of the arms [17]. When drugs are loaded onto the core of a star polymer, it is possible to compartmentally isolate them from the external environment through the shell, allowing for more stable retention of the unstable drugs. Moreover, multi-armed star polymers could assemble into higher-order architectures with auspicious physicochemical properties for developing drug delivery systems [18,19].

Herein, we developed star block copolymers with poly(furfuryl glycidol) (PFG) cores and biocompatible poly(ethylene glycol) (PEG) shells for drug delivery applications. PFG is a polymer material based on an epoxy with furfuryl groups, which enables the control of drug retention and release through the utilization of the Diels–Alder reaction. PFG-based DDS has been employed in various forms. For instance, microcapsules and nanoparticles utilizing an epoxy resin matrix derived from a furfuryl glycidyl ether have been developed, facilitating drug encapsulation and controlled release [20,21,22]. The star block copolymers were synthesized by anionic polymerization with initiators having four and six hydroxyl moieties for controlling the number of branches. The degree of polymerization of PFG and PEG segments was also controlled by the addition of monomers, and the behavior of the resulting star-shaped block copolymers was studied in an aqueous condition. Moreover, the furfuryl group in the core blocks could provide drug conjugating capability through the Diels–Alder reaction with maleimide functionalized drugs, such as those used in antibody drug conjugates [23].

Thus, we evaluated the ability to incorporate and release maleimide-functionalized fluorescent probes as model drugs by the Diels–Alder and retro Diels–Alder reactions, respectively. In addition, the ability of the polymers to circulate in the bloodstream was tested by intravital microscopy. Our results demonstrated the importance of increasing the number of arms and the length of the PEG blocks for stabilizing the star-shaped block copolymers in aqueous conditions. The PFG-PEG star-shaped block copolymers showed high loading capacity and long circulation in the blood, supporting their potential as drug delivery systems for in vivo use. 

## 2. Materials and Methods

### 2.1. Materials

1,1,2,2-Tetrakis(4-hydroxyphenyl)ethane, propyl-maleimide, dehydrated benzene, dimethyl Sulfoxide (Super Dehydrated), Penicillin–Streptomycin Solution (×100), and sodium hydroxide were purchased from Fujifilm Wako Pure Chemical (Osaka-shi, Osaka, Japan). Myo-Inositol, a furfuryl glycidyl ether, the phosphazene base P4-*t*-Bu 0.8 M in hexane, 1-*tert*-butyl-4,4,4-tris(dimethylamino)-2,2-bis[tris(dimethylamino)-phosphoranylide-nimino]-2λ5,4λ5-catenadi(phosphazene), dichloromethane, 6-maleimidohexanoic acid, DMSO-d_6_, and CDCl_3_, and Dulbecco’s modified eagle’s medium (DMEM)-high glucose were purchased from Sigma-Aldrich (St. Louis, MO, USA). Ethylene oxide was purchased from 3M Japan (Shinagawa-ku, Tokyo, Japan). Alexa Fluor 488 C5 Maleimide was purchased from Thermo fisher scientific (Waltham, MA, USA). The Sulfo-Cyanine5 NHS ester was purchased from Lumiprobe (Cockeysville, MD, USA). Eight arm-PEG-NH_2_ was purchased from Creative PEGworks (Chapel Hill, NC, USA). *N*, *N*-Dimethylformamide was purchased from Tokyo Chemical Industry (Tokyo, Japan). *N*, *N*-Dimethylformamide (dehydrated) was purchased from Kanto Chemical (Chuo-ku, Tokyo, Japan). D-PBS (-) (1×) (PBS) was bought from Nacalai Tesque (Nakagyo-ku, Kyoto, Japan). Hoechst 33,342 solution and 2-Morpholinoethanesulfonic acid monohydrate (MES) were purchased from Dojindo Laboratories (Mashiki-machi, Kumamoto, Japan). Human pancreatic adenocarcinoma BxPC3 cells were purchased from the American Type Culture Collection (Manassas, VA, USA). Fetal Bovine Serum (FBS) was purchased from Biosera (Kansas City, MO, USA). Spectra/Por (molecular weight cut-off (MWCO): 3.5 kDa) was purchased from Spectrum Laboratories (Rancho Dominguez, CA, USA). 

### 2.2. Measurements

Molecular weight and molecular weight distribution were measured by high-performance liquid chromatography (HPLC; EXTREMA, JASCO, Tokyo, Japan) with an intelligent sampler (AS-2051Plusm, JASCO, Tokyo, Japan), intelligent HPLC pump (PU-2080 Plus, JASCO, Tokyo, Japan), dynamic mixer (MX-2080-32, JASCO, Tokyo, Japan), 4-Line Degasser (DG-2080-54, JASCO, Tokyo, Japan), intelligent column oven (CO-2065 Plus, JASCO, Tokyo, Japan), multiwavelength detector (MD-2015 Plus, JASCO, Tokyo, Japan), intelligent UV/VIS detector (UV-2070 Plus, JASCO, Tokyo, Japan), and an LC-NetⅡ/ADC (JASCO, Tokyo, Japan). The chemical composition of the synthesized polymers was evaluated by ^1^H nuclear magnetic resonance spectroscopy (JMTC-400/54/SS, JEOL, Tokyo, Japan). The radius of the molecules was evaluated by dynamic light scattering (Zetasizer Nano-ZS, Malvern Instruments, Malvern, United Kingdom) using a laser with a wavelength of 532 nm. The images of the cells were obtained by confocal laser scanning microscopy (CLSM; LSM 780, Carl Zeiss, Germany). Intravital CLSM was performed using a Nikon A1R (Nikon, Japan).

### 2.3. Polymerization of the 4-Arm PFG-PEG

**Scheme 1:**
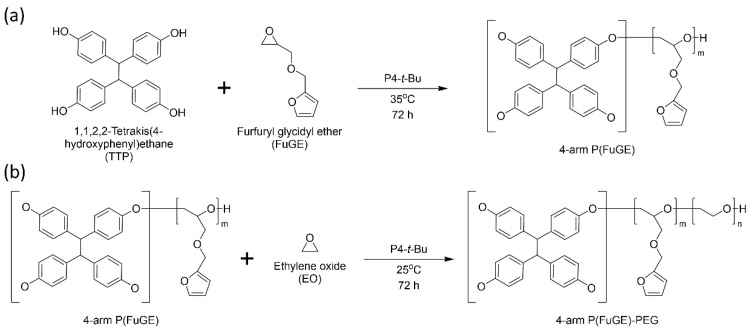
Polymerization of (**a**) 4-arm PFG, and (**b**) 4-arm PFG-PEG.

All the procedures were carried out under a dried argon atmosphere. The four-arm initiator 1,1,2,2-Tetrakis(4-hydroxyphenyl) ethane (TTP) (16 mg) was added to the two-neck flask and dehydrated by lyophilization with anhydrous benzene. Super-dehydrated DMF (10 mL) was added to dissolve TTP, and the phosphazene base P_4_-*t*-Bu (200 μL) was added by a syringe. A furfuryl glycidyl ether (FuGE) (220, 440, 880 μL) was injected by a syringe and stirred for 72 h at 35 °C. The samples were purified by dialysis (MWCO: 3.5 kDa) against dichloromethane for 2 days. Then, a series of TTP-initiated 4-arm PFGs with different degrees of polymerization were obtained as brown oil after the evaporation of dichloromethane (Scheme 1a, Table 1). The molecular weight and polymerization degree (DP) of the obtained polymers were analyzed by ^1^H-NMR spectroscopy and gel permeation chromatography (GPC). The polymers were named based on the initiator and the degree of polymerization of the PFG block as follows: TTP-PFG10 for PFG blocks with 10 units, TTP-PFG20 for PFG blocks with 20 units, and TTP-PFG50 for PFG blocks with 50 units, per 1 arm. 

The obtained TTP-PFGs were dehydrated by lyophilization with anhydrous benzene in the two-neck flask. Then, super-dehydrated DMF was injected by a syringe to dissolve the TTP-PFGs. P_4_-*t*-Bu (200 μL) and EO were injected by a syringe (cooled with liquid nitrogen to be less than 0 °C) and stirred for 72 h at 25 °C. The reaction mixtures were purified by dialysis (MWCO: 3.5 kDa) against dichloromethane for 2 days. Then, a series of 4-arm PFG-PEGs were obtained after the removal of dichloromethane by evaporation (Scheme 1b). The amount of chemicals used in this reaction is summarized in Table 2. The molecular weight, DP, and hydrodynamic radius (in water and DMF) of the polymers were analyzed by GPC, ^1^H-NMR spectroscopy, and dynamic light scattering (DLS). The final polymers were named based on the initiator, the degree of polymerization of the PFG block, and the degree of polymerization of the PEG segment per 1 arm as follows: TTP-PFG10-PEG150, TTP-PFG10-PEG500, TTP-PFG10-PEG1000, TTP-PFG20-PEG70, TTP-PFG20-PEG90, TTP-PFG20-PEG430, TTP-PFG50-PEG60, and TTP-PFG50-PEG150, where the numbers indicate the degree of polymerization of each block. The chemical composition and hydrodynamic radius of the polymers are summarized in Table 3 and Table 4.

### 2.4. Polymerization of the 6-Arm PFG-PEG

**Scheme 2:**
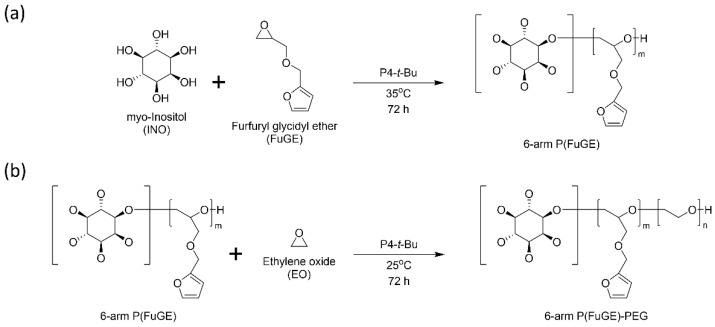
Polymerization of (**a**) 6-arm PFG, and (**b**) 6-arm PFG-PEG.

All the procedures were performed under a dried argon atmosphere. The 6-arm initiator myoinositol (INO) (6 mg) was added to the two-neck flask and dehydrated by lyophilization with anhydrous benzene. Super-dehydrated DMSO (3 mL) was poured into the system to dissolve INO. Then, P_4_-*t*-Bu (200 μL) was added by a syringe. FuGE (275 μL) was poured by a syringe and stirred for 72 h at 35 °C. The reaction mixtures were purified by dialysis against dichloromethane for 2 days (MWCO: 3.5 kDa). A 6-arm PFG was obtained as brown oil after evaporating dichloromethane (Scheme 2a, Table 1). The molecular weight and DP of the obtained polymers were analyzed by GPC and ^1^H-NMR spectroscopy. The polymer was named based on the initiator and the degree of polymerization of the PFG block per 1 arm as INO-PFG10, where the number indicates the degree of polymerization of the PFG block.

The obtained INO-PFG10 was dehydrated by lyophilization with anhydrous benzene in a two-neck flask. Then, super-dehydrated DMSO was injected by a syringe into the system to dissolve INO-PFG10. P_4_-*t*-Bu and EO were then injected into the system by a syringe (cooled with liquid nitrogen to be less than 0 °C) and stirred for 72 h at 25 °C. The reaction mixtures were purified by dialysis against dichloromethane for 2 days (MWCO: 3.5 kDa), and two 6-arm PFG-PEG star-shaped block copolymers were obtained after evaporating dichloromethane (Scheme 2b). The amount of chemicals used in this reaction is summarized in Table 2. The molecular weight, polymerization degree (DP), and hydrodynamic radius (in DI water and DMF) of the obtained polymers were analyzed by GPC, ^1^H-NMR spectroscopy, and DLS. The final polymers were named based on the initiator, the degree of polymerization of the PFG block, and the degree of polymerization of the PEG segment The polymer was named based on the initiator and the degree of polymerization of the PFG block per 1 arm as INO-PFG10, where the number indicates the degree of polymerization of the PFG block as follows: INO-PFG10-PEG60, TTP-PFG10-PEG120, where the numbers indicate the degree of polymerization of each block. The chemical composition and hydrodynamic radius of the polymers are summarized in Table 3 and Table 4.

### 2.5. Introduction of a Model Drug via a Diels–Alder Reaction

**Scheme 3:**
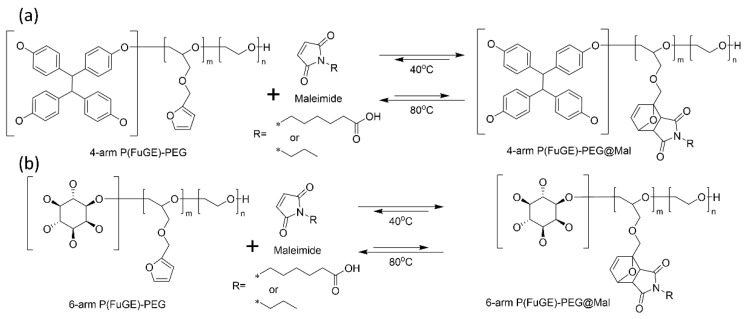
Model drug loading and release via a Diels–Alder reaction. (**a**) The 4-arm P(FuGE)-PEG. (**b**) The 6-arm P(FuGE)-PEG.

The introduction of model drugs bearing maleimide moieties, namely, *N*-propylmaleimide (pMal) and maleimidohexanoic acid (MalC), to 4/6-arm PFG-PEGs, was evaluated (Scheme 3). Star-shaped block copolymers (TTP-PFG50-PEG60 (427 mg), INO-PFG10-PEG120 (303 mg)), and pMal (835 mg, 334 mg, 251 mg) or MalC (1267 mg, 381 mg)) were mixed with dichloromethane (5 mL) and stirred for 120 h at 40 °C. The solutions were collected and purified by dialysis (MWCO: 3.5 kDa) against dichloromethane. The polymers were collected by evaporating the dichloromethane. The introduction amounts of pMal and MalC were evaluated by ^1^H-NMR. The hydrodynamic radius was determined by DLS in DMF in water and PBS at pH 8. Moreover, the time-dependent introduction of pMal in the polymers was evaluated using the star-shape block copolymer TTP-PFG50-PEG60. The TTP-PFG50-PEG60 (427 mg) and pMal (835 mg) were mixed in dichloromethane (5 mL) and stirred at 40 °C. Then, 1 mL of the reaction solution was collected after 24, 48, 72, 96, and 120 h. The sample was purified by dialysis in dichloromethane (MWCO: 3.5 kDa). The introduced amount of pMal in the star-shaped block copolymer was quantified by ^1^H-NMR. 

### 2.6. Release of Model Drugs by a Retro Diels–Alder Reaction

The time-dependent release of pMal from TTP-PFG50-PEG60-pMal via a retro Diels–Alder reaction was evaluated (Scheme 3). The TTP-PFG50-PEG60-pMal was placed in a flask with 5 mL of DMF and stirred at 80 °C. Then, 1 mL of the solution was sampled at 15, 30, 45, and 60 min, and dialyzed against dichloromethane (MWCO: 3.5 kDa). The pMal amount that remained conjugated in the polymer was quantified by ^1^H-NMR measurement. 

### 2.7. Preparation of Fluorescence-Labeled Polymers

Alexa Fluor 488-labeled 4-arm PFG-PEG (TTP-PFG50-PEG60-AF488) and Cy5-labeled 8-arm PEG (8-arm-PEG-Cy5) were synthesized by introducing Alexa Fluor 488 C5 Maleimide (AF488) and a sulfo-Cyanine 5-NHS ester (Cy5-NHS), respectively. Thus, TTP-PFG50-PEG60 (50 mg) and AF488 (840 μg) were mixed in dichloromethane (5 mL) at 40 °C for 120 h. The solution was purified by dialysis (MWCO: 3.5 kDa) against dichloromethane. The dichloromethane was then evaporated and the TTP-PFG50-PEG60-AF488 powder was obtained by lyophilization from benzene. The 8-arm PEG-NH_2_ (40 kDa) (400 mg) and Cy5-NHS (7.78 mg) were mixed in an MES buffer (5 mL) and stirred for 12 h. The solution was purified by dialysis (MWCO: 3.5 kDa) against water. The 8-arm-PEG-Cy5 was collected after lyophilization. 

### 2.8. In Vitro Cellular Uptake

The cellular uptake of TTP-PFG50-PEG60-AF488 and 8-arm-PEG-Cy5 was evaluated by CLSM. BxPC3 cells (5 × 10^4^ cells/300 μL) were seeded in 8-well chamber slide glasses. After pre-incubation for 24 h, the medium was gently removed and the solution of TTP-PFG50-PEG60-AF488, and 8-arm-PEG-Cy5 (both 1 mg/mL in medium) was added to the cells. After incubation for 24 h, the medium was gently removed and washed with PBS twice. Hoechst solution (2 μg/mL in PBS) was added, and the cells were incubated for 10 min at 37 °C. Then, the cells were gently washed with PBS three times and imaged by CLSM using an Ar laser (excitation: 488 nm; emission: 520 nm) for TTP-PFG50-PEG60-AF488 and a He/Ne laser (excitation: 630 nm; emission: 670 nm) for 8-arm-PEG-Cy5.

### 2.9. Blood Circulation

Female 7-week-old BALB/c mice anesthetized with 2% isoflurane inhalation were placed on a 37 °C heat stage under a Nikon A1R CLSM attached to an upright ECLIPSE Ni-E equipped with a CFI Plan Apo Lambda 20×/0.75 objective lens. Transitions in fluorescent intensity of the right earlobe veins were recorded. TTP-PFG50-PEG60-AF488 (3 mg/mL in a 100 μL volume) and 8-arm-PEG-Cy5 (0.5 mg/mL in a 100 μL volume) were injected concomitantly in a 30 s period from the tail vein starting from 10 s after the beginning of the recording. Recordings were performed continuously for 10 min followed by 5 min interval imaging for 7 h. For excitation, 480 nm and 640 nm lasers were used at an intensity of 5% and 20% (less than 1.5 mW and 2 mW, respectively). For detection, 500–550 nm and 662–737 nm emission filters were used. Average fluorescent intensity transitions were measured for the region of interest set in the earlobe vein for each recording. An increase in fluorescent intensity was calculated by subtracting the background before sample injection. The maximum increase in fluorescent intensity was defined as 100%.

## 3. Results and Discussion

### 3.1. Synthesis and Characterization of the 4/6-Arm PFG-PEG

Ring-opening polymerization of glycidol and ethylene oxide is usually performed using potassium naphthalene for deprotonation of the hydroxy group of initiator alcohol. However, a stronger base was required for initiating multivalent hydroxy groups equally with high efficiency. Therefore, we applied a fourth generation phosphazene base, which presents a nitrogen basic center double bonded to pentavalent phosphorus. For the 4-arm and 6-arm initiators, we used 1,1,2,2-Tetrakis(4-hydroxyphenyl) ethane and myoinositol, respectively. All 4- and 6-arm PFGs, i.e., TTP-PFG10, TTP-PFG20, TTP-PFG50, and INO-PFG10, displayed monodispersed gel permeation chromatograms (Supplementary Figures S1–S3 and S12), which suggests the successful polymerization of branched PFGs with a comparable length of branches. Because the molecular weight of branched polymers cannot be evaluated by GPC with a linear standard, the DP of branched polymers was estimated by ^1^H-NMR (Supplementary Figures S1–S3 and S12). All NMR spectra indicate the appropriate DP for each star-shaped polymer. The obtained DPs are summarized in Table 3.

Second, polymerization of the EO from PFGs was performed using the phosphazene base. The GPC results of these polymers indicate monodisperse distribution (Supplementary Figures S4–S11, S13 and S14). The molecular weight and DP of the obtained 4/6-arm PFG-PEGs were evaluated by ^1^H-NMR (Supplementary Figures S4–S11, S13 and S14). All the polymers showed the expected DP (Table 3).

### 3.2. Hydrodynamic Radius Measurement of the 4/6-Arm PFG-PEG

The hydrodynamic radii of 4/6-arm PFG-PEGs were measured by DLS in DMF and water (Table 4). In DMF, the hydrodynamic radii of the star-shaped block copolymers were smaller than 7 nm. However, the hydrodynamic radii measured in water increased, ranging from 20 to 100 nm. This solvent-dependent change in hydrodynamic radii could be explained by the self-organization of the star polymers in water probably being mediated by the hydrophobic core of the star polymers, i.e., the furfuryl-bearing blocks and the initiator center. The elevated values of polydispersity indices (PDIs) observed in DMF could be attributed to the small size of the star polymers, which makes accurate measurement using DLS challenging.

Assuming that the linear polymer is free-rotating and has appropriate solubility in the solvent (*θ*-solvent), the squared radius of inertia <*Rg*> can be calculated by the following equation:

<*Rg*>^2^ = *nb*^2^/6 (1)

where *n* is the number of atoms from end to end and *b* is the distance between atoms in the main chain in nm. Moreover, the ratio “*g*” of the squared radius of inertia of a star-shaped block copolymer and linear polymer, when it is in the *θ* solvent, can be approximated by the following equation [24]:

g = <Rg>_star_^2^/<Rg>_linear_^2^ = (3p − 2)/p^2^ (2)

where *p* is the number of branches. These equations have been applied to various star copolymers, such as polystyrene, polyisoprene, and polybutadiene, and the results were consistent with theoretical values in theta and good solvents at *p* ≤ 6 [25,26,27]. Since the branched numbers in our systems are four and six, Equation (2) can be applied. Additionally, the branched copolymers are also thought to have a rigid sphere-like structure as the number of arms increases, so the ratio of hydrodynamic radius to squared inertia radius, which is an indicator of geometric factors, has also been reported as the following Equation (3):

ρ = <Rg>/<Rh>(3)

where *Rh* is the fluid dynamics radius in nm. The *ρ* values of rigid spheres and linear polymers are calculated by Equation (3) as 0.775 and 1.479, respectively [28,29]. It has also been estimated that the *ρ* value of the branched polymer with 18 arms was about 0.8–0.9 [30]. Thus, the *ρ* value is approximated to 1 for simplifying the calculation for 4/6-arm PFG-PEGs. The calculated *Rh* is summarized in Table 4.

Comparing the measured hydrodynamic radii in DMF and the calculated fluid dynamics radii of the star-shaped block copolymers, we found that both radii are comparable, which support the presence of 4/6-arm PFG-PEGs as single molecules in DMF. On the other hand, the size of the polymers in water was much larger, suggesting the association of the molecules. Thus, 4-arm PFG-PEGs (TTP series) showed hydrodynamic radii around 40–90 nm in water. The radii of INO-PFG10-PEG60 and INO-PFG10-PEG120 were around 20 nm in water, which is much smaller than 4-arm PFG-PEGs. This difference may be due to the increased steric repulsion from the PEG branches in 6-arm PFG-PEGs. 

### 3.3. Time-Dependent Loading and the Release of a Maleimide-Bearing Model Drug

**Figure 1:**
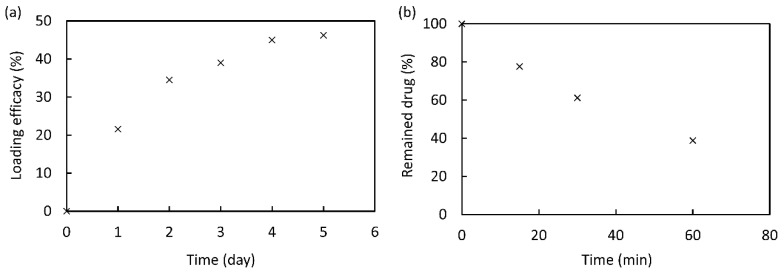
(**a**) Time-dependent loading of maleimide to TTP-PFG50-PEG60 via a Diels–Alder reaction at 40 °C. (**b**) Time-dependent release of maleimide from TTP-PFG50-PEG60-pMal via a retro–Diels–Alder reaction at 80 °C.

The time-dependent loading of pMal as a model drug to the star-shaped block copolymers via a Diels–Alder reaction was evaluated in TTP-PFG50-PEG60. The introduction rate was calculated from the amount of maleimide introduced into the furfuryl group per polymer molecule. The reaction reached equilibrium at 4 days, displaying a 40% introduction of pMal (Figure 1a). These results support the ability of the star-shaped block copolymers for loading maleimide-bearing prodrugs in their inner compartment. 

The retro Diels–Alder reaction was also followed in time. TTP-PFG50-PEG60-pMal was incubated at 80 °C and the released pMal was followed by ^1^H-NMR. The results show that 60% of pMal can be detached from the polymer through a retro Diels-Alder reaction within 60 min (Figure 1b). The release of pMal occurred rapidly, which suggests the potential of the system for generating temperature-responsive nanocarriers for therapeutic objectives. For instance, radiofrequency or microwave ablation treatments apply temperatures exceeding 80 °C to the tissues [31]. Thus, these thermo-responsive systems have the potential for being used in combination with these therapeutic approaches.

### 3.4. Loading of Maleimide-Bearing Model Drugs

The loading of different types of maleimide-bearing model drugs was evaluated in several star-shaped block copolymers. The model drugs include hydrophobic pMal and hydrophilic MalC. The conjugation was tested in TTP-PFG50-PEG60 and INO-PFG10-PEG120 (Table 5). After a 120 h reaction, the introduction rate went from 67 up to 96% of the core furfuryl groups, which supports the high loading capacity of the star-shaped block copolymers. Notably, in TTP-based systems, MalC had higher drug introduction efficiency than pMal. 

The changes in the size of the star-shaped block copolymers before and after the Diels–Alder reaction were evaluated by DLS (Table 6). When pMal was loaded, an increase in the size was observed for all the star-shaped block copolymers, which was considered to be due to the volume increase in the core and the enhanced association on the block copolymers. Regarding MalC, the sizes of TTP-PFG50-PEG60 and INO-PFG10-PEG120 increased, probably because of the abovementioned hypothesis. This suggests that the introduction of hydrophilic MalC may reduce the hydrophobicity of the core, and thereby, the association of the copolymers. Notably, the size of all the MalC-loaded star-shaped block copolymers was significantly decreased to less than 10 nm in PBS at pH 8. The reduced size of the block copolymers in basic conditions could be related to the generation of negatively charged carboxylate units in MalC, as suggested by a negatively charged zeta potential, which prevented the association of the block copolymers by electrostatic repulsion.

### 3.5. Cellular Uptake Evaluation

**Figure 2:**
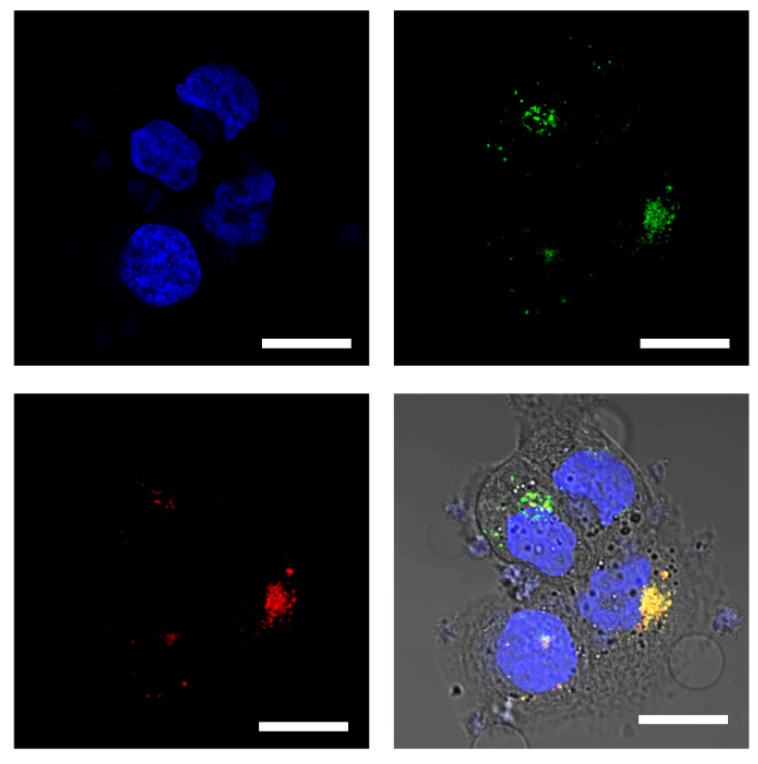
Cellular uptake of TTP-PFG50-PEG60-AF488 in BxPC3 cells (blue: nuclei; green: TTP-PFG50-PEG60-AF488; red: the 8-arm PEG-Cy5; yellow: co-localization of TTP-PFG50-PEG60-AF488 and the 8-arm-PEG-Cy5). Scale bar: 20 μm.

The cellular uptake behavior of fluorescently modified TTP-PFG50-PEG60 was studied in human pancreatic adenocarcinoma BxPC3 cells (Figure 2). The polymer was labeled with maleimide, Alexa Fluor 488 (TTP-PFG50-PEG60-AF488). The star polymer displayed a comparable uptake and distribution of the Cy5-conjugated 8-arm PEG, which was used as the control. Moreover, high co-localization of TTP-PFG50-PEG60-AF488 and the 8-arm-PEG-Cy5 was observed (Figure 2; yellow color). This observation indicates the potential of the star-shaped block copolymers for the intracellular delivery of drugs.

### 3.6. Blood Circulation

**Figure 3:**
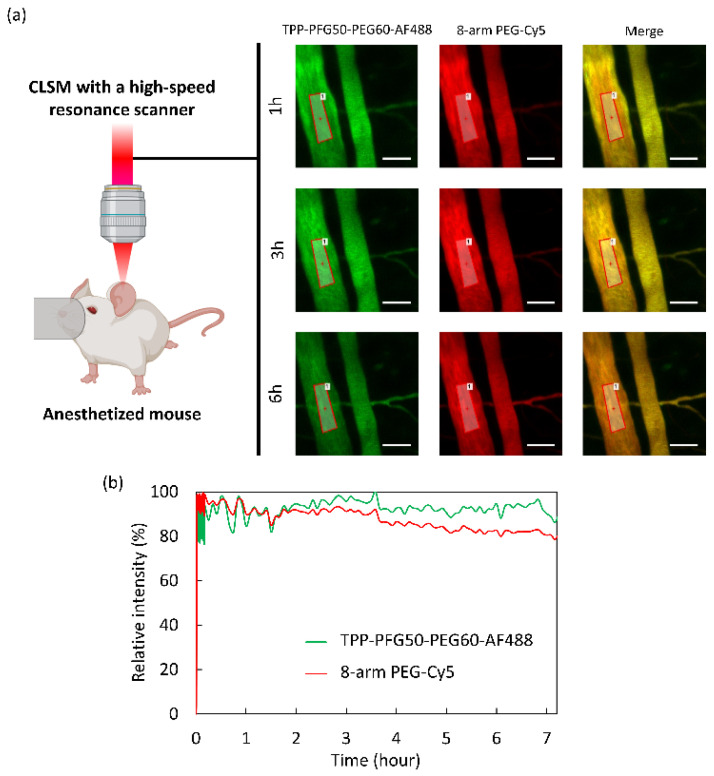
The blood circulation of TTP-PFG50-PEG60-AF488 and the 8-arm PEG-Cy5. (**a**) IVRT-CLSM images in the earlobe of a mouse. Scale = 50 μm. (**b**) Time-dependent profile of the fluorescent intensity in the bloodstream obtained from the region of interest in (**a**).

The blood circulation of the star-shaped block copolymers was studied in a mouse by intravital real-time CLSM (IVRT-CLSM). In this experiment, we intravenously co-injected TTP-PFG50-PEG60-AF488 and the 8-arm-PEG-Cy5, which have almost the same molecular weight, i.e., around 40 kDa, and followed their behavior in the blood vessels in the earlobe skin of mice. Figure 3a shows the images recorded in the earlobes at 1, 3, and 6 h after injection, and Figure 3b shows the quantification of the fluorescent signals. TTP-PFG50-PEG60-AF488 displays long blood circulation, which was comparable to that of the 8-arm-PEG-Cy5, and the amount in blood after 6 h exceeded 80% of the initial concentration in blood. These results support the ability of the star-shaped block copolymers to generate long-circulating nanocarriers for in vivo application. 

## 4. Conclusions

In this study, we synthesized 4- and 6-armed star-shaped block copolymers that consist of a PFG core for a Diels–Alder reaction-based drug conjugation and a PEG shell for biocompatibility. The star-shaped block copolymers can efficiently load maleimide-bearing molecules through the Diels–Alder reaction. Moreover, the drugs can be rapidly released *via* a retro Diels–Alder reaction triggered by heating. The star-shaped block copolymers also exhibited prolonged blood circulation and the potential for intracellular drug delivery. As the end-groups of the PEG chains of the star polymers can be further modified with ligands, such as peptides or antibodies, we anticipate the potential for these nanocarriers to generate actively targeted formulations with improved delivery ability.

## Figures and Tables

**Table 1:** The amount of initiator, solvent, P_4_-*t*-Bu, and PFG.

Sample	Initiator (μmol)	Solvent (mL)		P_4_-*t*-Bu (μmol)	FuGE (mmol)
TTP-PFG10	40	DMF	(2.5)	160	1.6
TTP-PFG20	40	DMF	(5)	160	3.2
TTP-PFG50	40	DMF	(10)	160	6.4
INO-PFG10	33	DMSO	(3)	200	2.0

**Table 2:** The amount of macro initiator, solvent, P_4_-*t*-Bu, and EO.

Sample	Macro Initiator (μmol)		Solvent (mL)		P4-*t*-Bu (μmol)	EO (mmol)
TTP-PFG10-PEG150	TTP10	40	DMF	(5)	160	9.60
TTP-PFG10-PEG500			DMF	(10)	160	19.2
TTP-PFG10-PEG1000			DMF	(20)	160	38.4
TTP-PFG20-PEG70	TTP20	40	DMF	(3.5)	160	6.4
TTP-PFG20-PEG90			DMF	(7)	160	12.8
TTP-PFG20-PEG430			DMF	(10)	160	25.6
TTP-PFG50-PEG60	TTP50	40	DMF	(3.5)	160	6.40
TTP-PFG50-PEG150			DMF	(7)	160	12.8
INO-PFG10-PEG60	INO10	33	DMSO	(6)	200	12
INO-PFG10-PEG120			DMSO	(13)	200	24

**Table 3:** Degree of polymerization and molecular weight of 4/6-arm PFG-PEG block copolymers.

Sample	Aimed DP		Evaluated DP ^a^		Mw ^b^	
	PFG	PEG	PFG	PEG	PFG (kDa)	PFG-PEG (kDa)
TTP-PFG10-PEG150	10	150	10.9	154	7.11	34.3
TTP-PFG10-PEG500		500		539		102
TTP-PFG10-PEG1000		1000		1060		194
TTP-PFG20-PEG70	20	70	24.2	71.2	15.3	27.9
TTP-PFG20-PEG90		90		93		31.7
TTP-PFG20-PEG430		430		430		91.1
TTP-PFG50-PEG60	40	60	51.1	61.5	31.9	42.7
TTP-PFG50-PEG150		150		151		58.5
INO-PFG10-PEG60	10	60	13.7	66	12.9	30.3
INO-PFG10-PEG120		120		122		45.1

^a^ Determined by ^1^H-NMR spectroscopy with CDCl_3_ or DMSO-d6; ^b^ determined by GPC using DMF with 10 mM lithium chloride (LiCl) and calculated using poly(ethylene glycol) (PEG) standards.

**Table 4:** Hydrodynamic radii of 4/6-arm PFG-PEG block copolymers.

Sample	Average Hydrodynamic Radius in Water ± S.D. (nm) ^a^	PDI	Average Hydrodynamic Radius in DMF ± S.D. (nm) ^b^	PDI	Rh (nm) ^c^
TTP-PFG10-PEG150	31 ± 1	0.104	-		5.33
TTP-PFG10-PEG500	30 ± 1	0.251	-		9.52
TTP-PFG10-PEG1000	23 ± 3	0.288	-		12.4
TTP-PFG20-PEG70	76 ± 4	0.236	5 ± 1	0.070	4.00
TTP-PFG20-PEG90	52 ± 7	0.411	4 ± 1	0.069	4.42
TTP-PFG20-PEG430	42 ± 1	0.213	5 ± 1	0.226	8.94
TTP-PFG50-PEG60	85 ± 1	0.146	5 ± 1	0.229	4.42
TTP-PFG50-PEG150	50 ± 2	0.179	5 ± 1	0.301	5.50
INO-PFG10-PEG60	23 ± 1	0.295	4 ± 1	0.451	3.64
INO-PFG10-PEG120	22 ± 1	0.371	6 ± 1	0.856	4.96

^a^ Determined by DLS with deionized-water (*n* = 3); ^b^ determined by DLS with DMF (*n* = 3); ^c^ calculated by formulas (1)–(3).

**Table 5:** Introduction number and ratio of maleimide as a model drug for 4/6-arm PFG-PEG block copolymers.

Sample	No. of Furfuryl Units	Introduction No. ^a^		Introduction Ratio ^a^	
		pMal	MalC	pMal (%)	MalC (%)
TTP-PFG50-PEG60	204	137	196	67.2	96.1
INO-PFG10-PEG120	82	71	71	86.6	86.6

^a^ Determined by ^1^H-NMR spectroscopy in DMSO-d6.

**Table 6:** Hydrodynamic radii of multi-arm PFG-PEGs after a Diels–Alder reaction ^a^.

Sample	Initial Radius in Water (nm)	Radius after pMal in Water (nm)	Radius after MalC in Water (nm)	After MalC in PBS at pH 8 (nm)
TTP-PFG50-PEG60	85	130	96	2.28
INO-PFG10-PEG120	22	27	25	4.51

^a^ Determined by DLS in deionized water or PBS.

## Data Availability

Not applicable.

## Supplementary Materials

The following supporting information can be downloaded at: https://www.mdpi.com/article/10.3390/polym15122626/s1, Figure S1: ^1^H-NMR spectrum (Solvent; CDCl_3_) and GPC curve of TTP-PFG10; Figure S2: ^1^H-NMR spectrum (Solvent; CDCl_3_) and GPC curve of TTP-PFG20; Figure S3: ^1^H-NMR spectrum (Solvent; CDCl_3_) and GPC curve of TTP-PFG50; Figure S4: ^1^H-NMR spectrum (Solvent; DMSO-d6) and GPC curve of TTP-PFG10-PEG150; Figure S5: ^1^H-NMR spectrum (Solvent; DMSO-d6) and GPC curve of TTP-PFG10-PEG500; Figure S6: ^1^H-NMR spectrum (Solvent; DMSO-d6) and GPC curve of TTP-PFG10-PEG1000; Figure S7: ^1^H-NMR spectrum (Solvent; DMSO-d6) and GPC curve of TTP-PFG20-PEG70; Figure S8: ^1^H-NMR spectrum (Solvent; DMSO-d6) and GPC curve of TTP-PFG20-PEG90; Figure S9: ^1^H-NMR spectrum (Solvent; DMSO-d6) and GPC curve of TTP-PFG20-PEG430; Figure S10: ^1^H-NMR spectrum (Solvent; DMSO-d6) and GPC curve of TTP-PFG50-PEG60; Figure S11: ^1^H-NMR spectrum (Solvent; DMSO-d6) and GPC curve of TTP-PFG50-PEG150; Figure S12: ^1^H-NMR spectrum (Solvent; DMSO-d6) and GPC curve of INO-PFG10; Figure S13: ^1^H-NMR spectrum (Solvent; DMSO-d6) and GPC curve of INO-PFG10-PEG60; Figure S14: ^1^H-NMR spectrum (Solvent; DMSO-d6) and GPC curve of INO-PFG10-PEG120.
