# Supplementary material for: Multi-Armed Star-Shaped Block Copolymers of Poly(ethylene glycol)-Poly(furfuryl glycidol) as Long Circulating Nanocarriers
Source: Polymers (Basel). 2023 Jun 9;15(12):2626. doi: 10.3390/polym15122626 (PMC10300759; doi:10.3390/polym15122626)
Supplement: Supplementary file 1 [file polymers-15-02626-s001.zip › polymers-2314500-supplementary.pdf]

Multi-Armed Star Block Copolymers of Poly(ethylene glycol)-Poly(Furfuryl Glycidol) as Long Circulating Nanocarriers

Yasuhiro Nakagawa[1,2], Kotaro Ushidome[1], Keita Masuda[1], Kazunori Igarashi[3], Yu Matsumoto[3] Tatsuya Yamasoba[3], Yasutaka Anraku [1], Madoka Takai[1], and Horacio Cabral[1*]

1. Graduate School of Engineering, The University of Tokyo, 7-3-1 Hongo, Bunkyo-ku, Tokyo 113-8656, Japan.
2. School of Materials and Chemical Technology, Tokyo Institute of Technology, 2-12-1 Ookayama, Meguro-ku, Tokyo, Japan.
3. Department of Otorhinolaryngology and Head and Neck Surgery, Graduate School of Medicine and Faculty of Medicine, The University of Tokyo, 7-3-1 Hongo, Bunkyo-ku, Tokyo, Japan.

*Corresponding author
horacio@bmw.t.u-tokyo.ac.jp; Tel.: +81-3-5841-7138

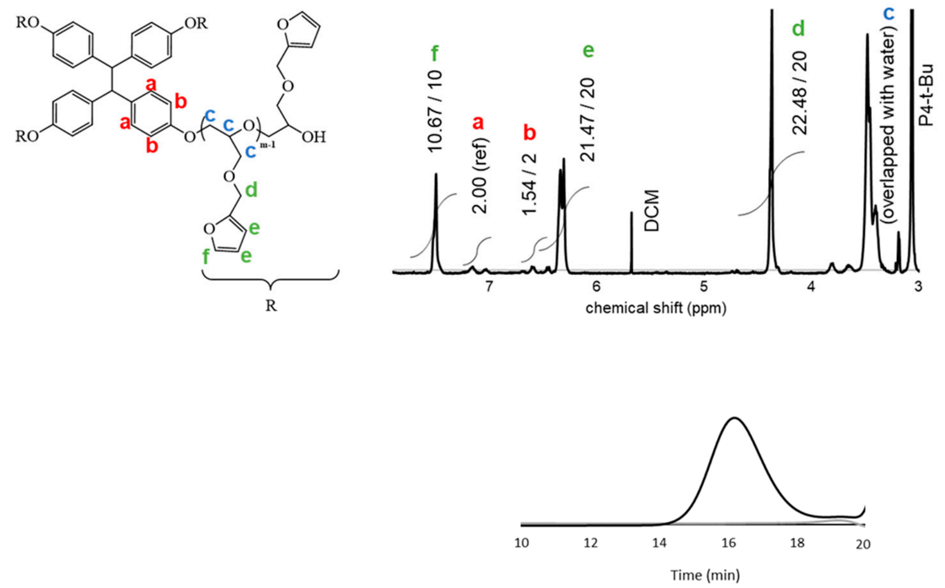

Figure S1. ^{1}H-NMR spectrum (Solvent; $CDCl_3$) and GPC curve of TTP-PFG10

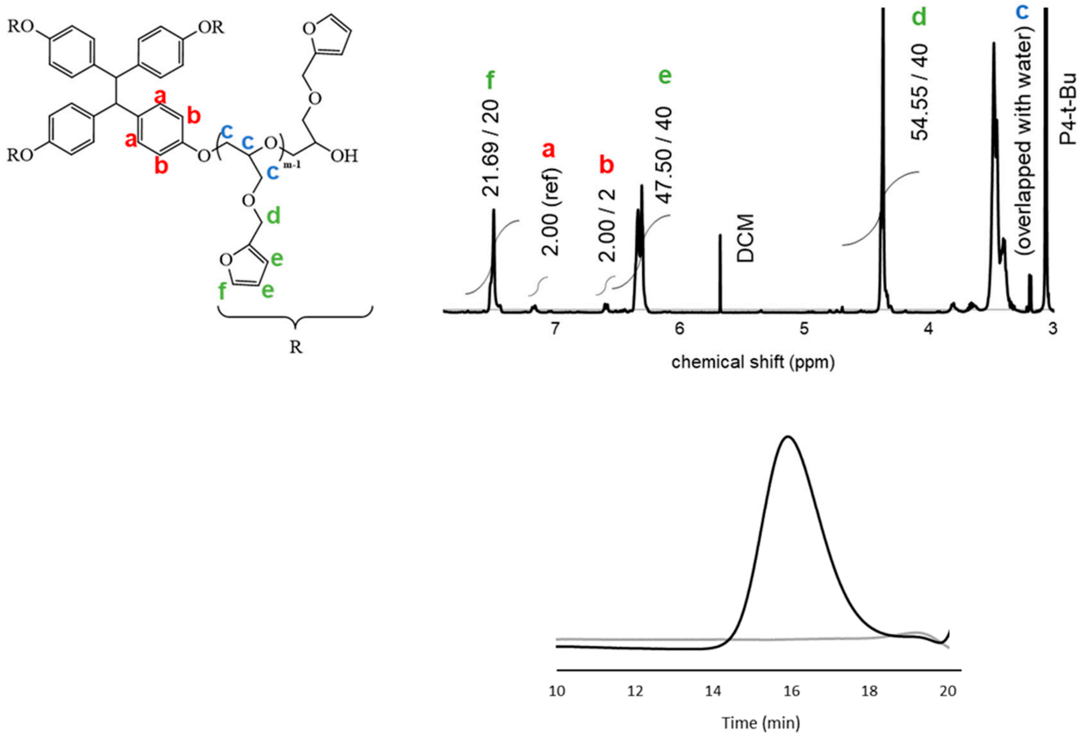

Figure S2. ^{1}H-NMR spectrum (Solvent; $CDCl_3$) and GPC curve of TTP-PFG20

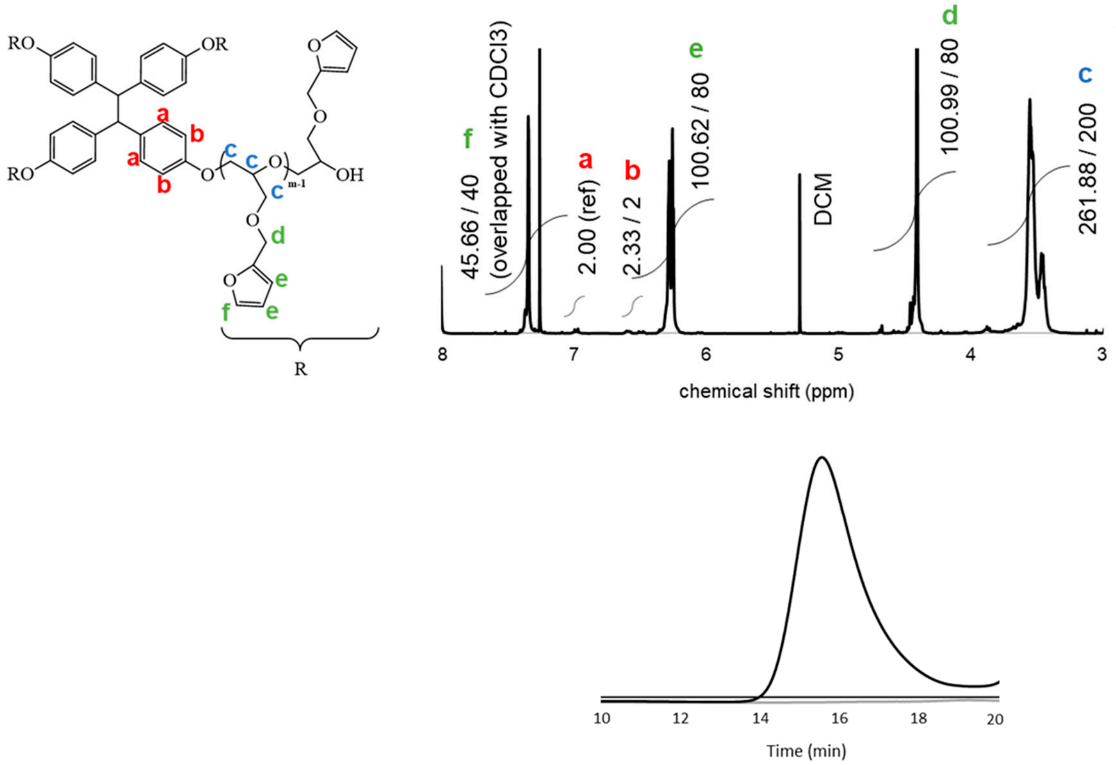

Figure S3. ^{1}H-NMR spectrum (Solvent; $CDCl_3$) and GPC curve of TTP-PFG50

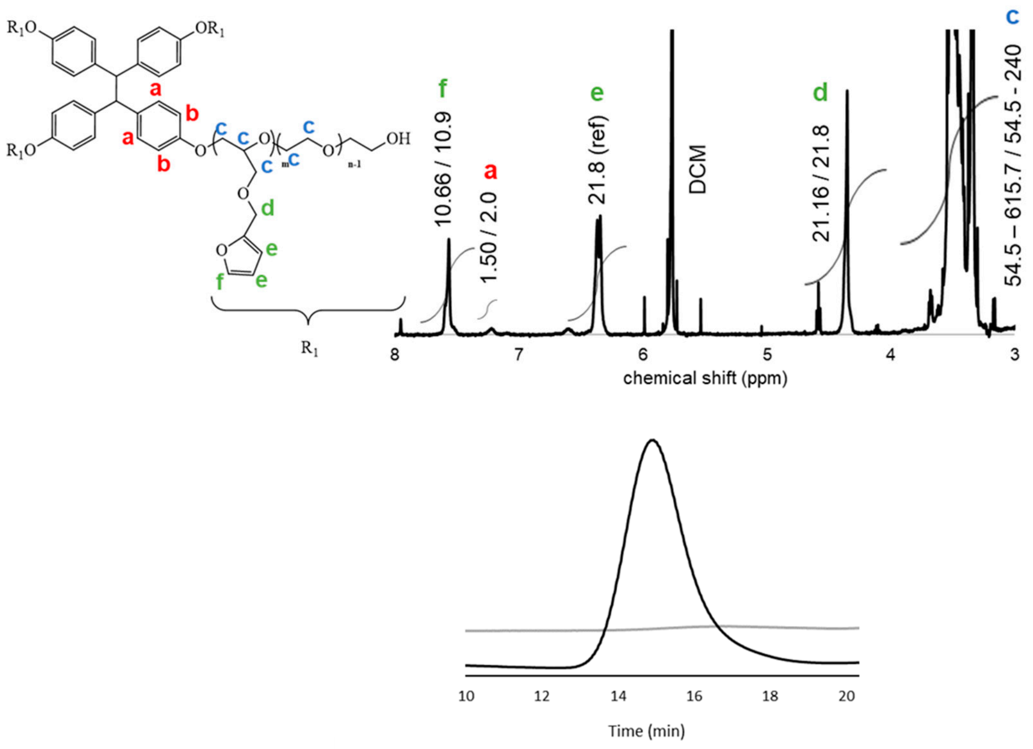

Figure S4. ^{1}H-NMR spectrum (Solvent; DMSO-d6) and GPC curve of TTP-PFG10-PEG150

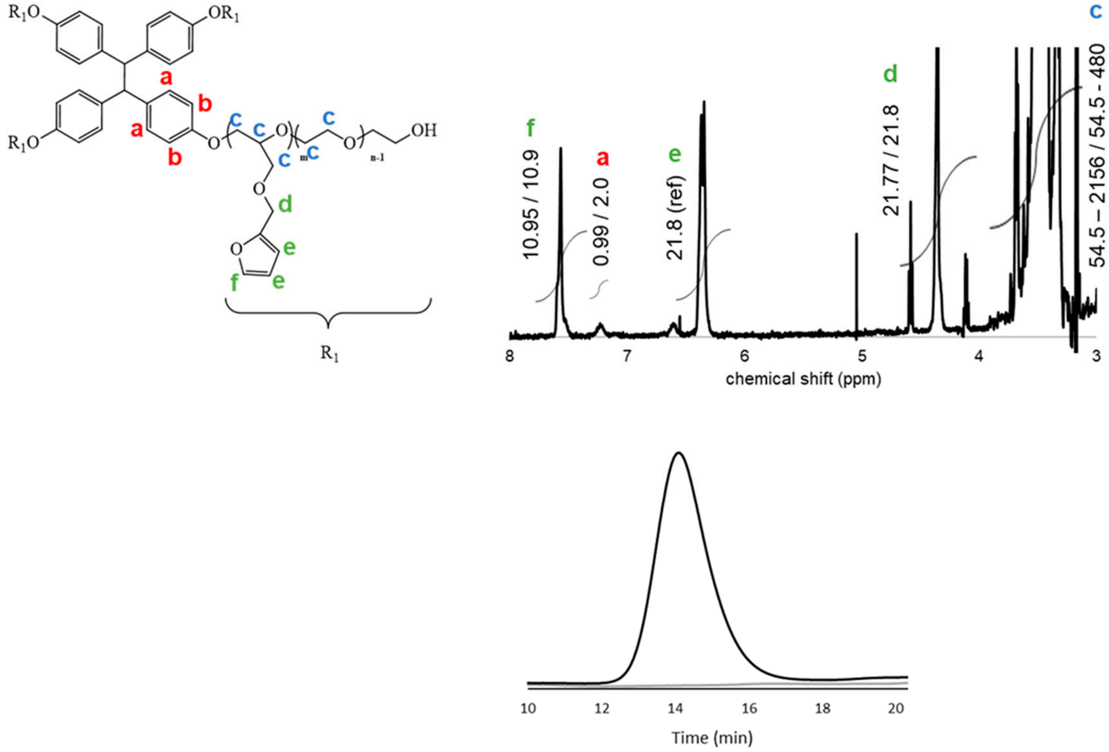

Figure S5. ^{1}H-NMR spectrum (Solvent; DMSO-d6) and GPC curve of TTP-PFG10-PEG500

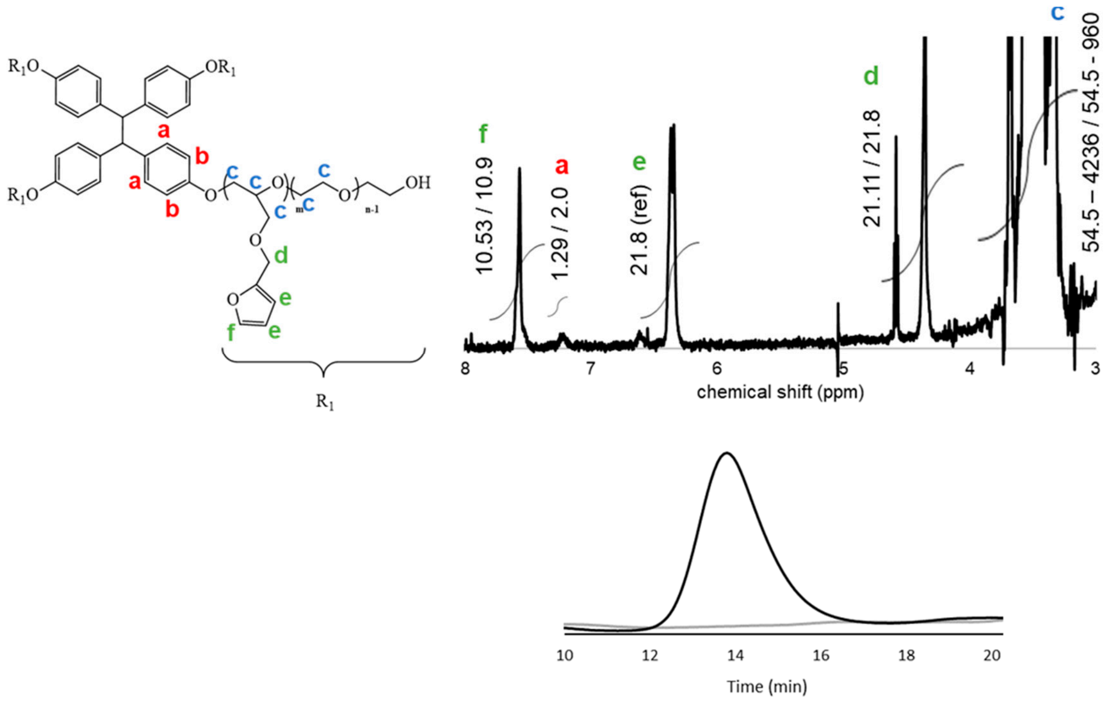

Figure S6. ^{1}H-NMR spectrum (Solvent; DMSO-d6) and GPC curve of TTP-PFG10-PEG1000

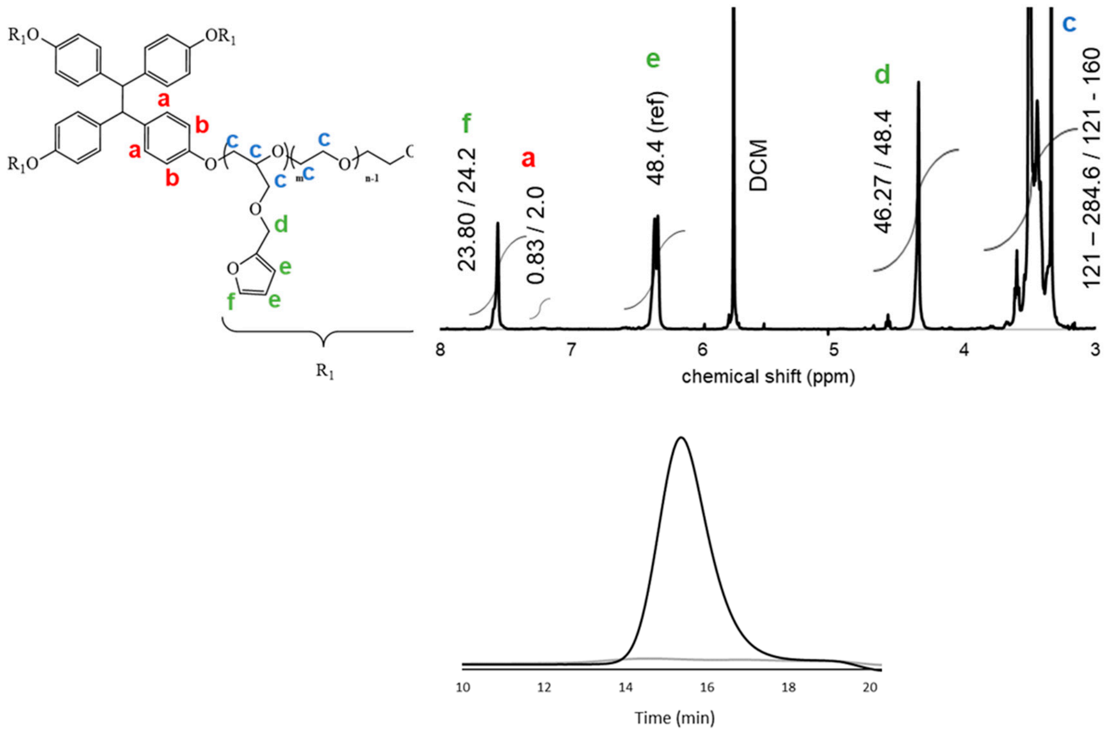

Figure S7. ^{1}H-NMR spectrum (Solvent; DMSO-d6) and GPC curve of TTP-PFG20-PEG70

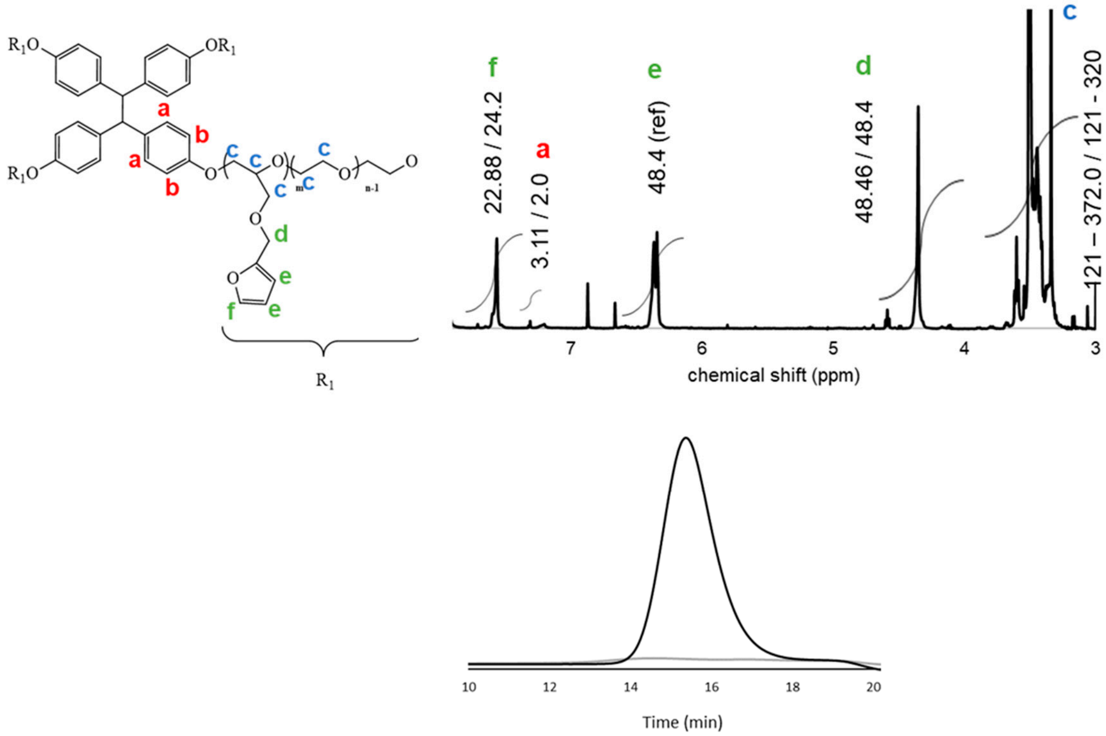

Figure S8. ^{1}H-NMR spectrum (Solvent; DMSO-d6) and GPC curve of TTP-PFG20-PEG90

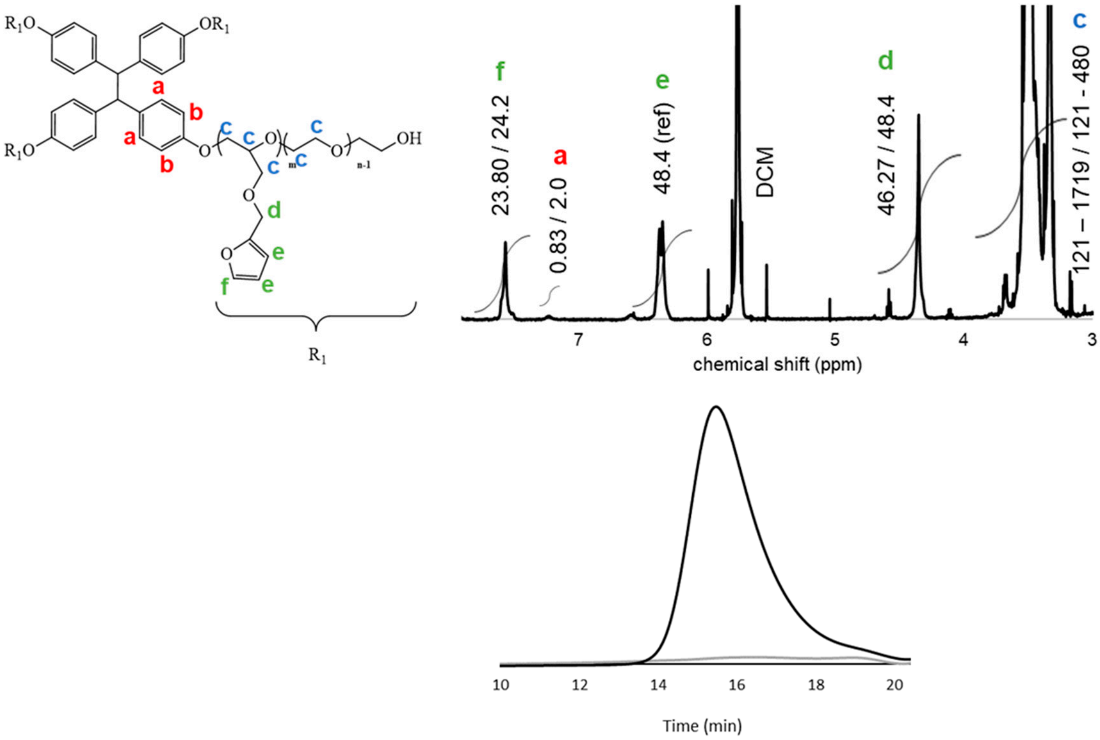

Figure S9. ^{1}H-NMR spectrum (Solvent; DMSO-d6) and GPC curve of TTP-PFG20-PEG430

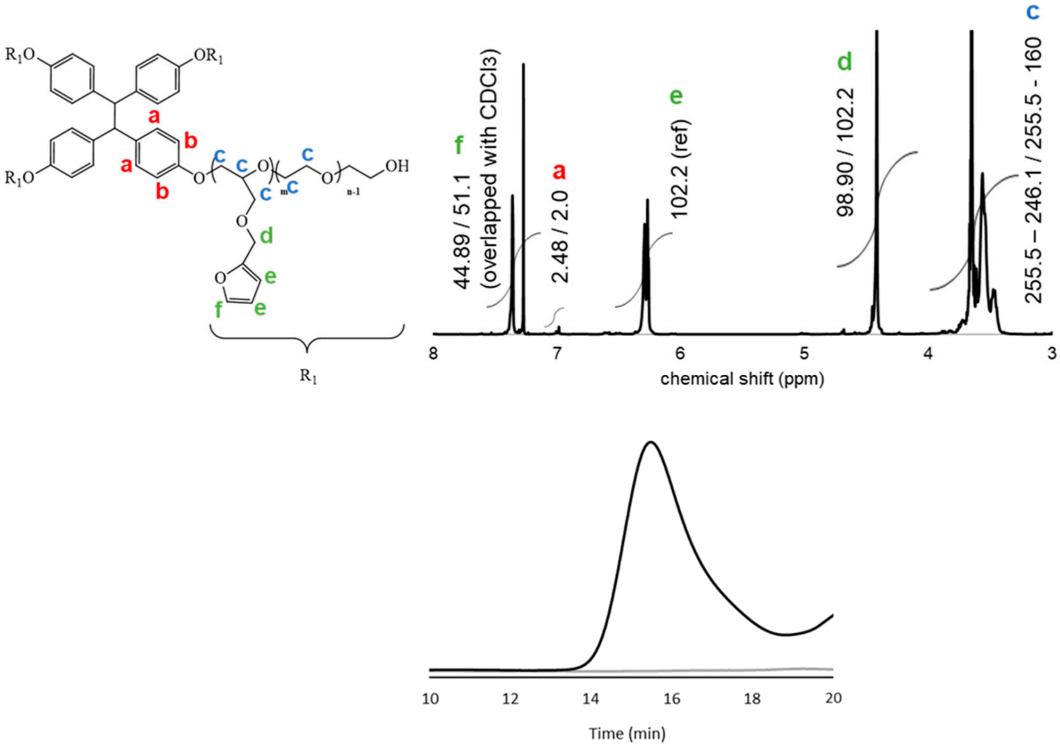

Figure S10. ^{1}H-NMR spectrum (Solvent; DMSO-d6) and GPC curve of TTP-PFG50-PEG60

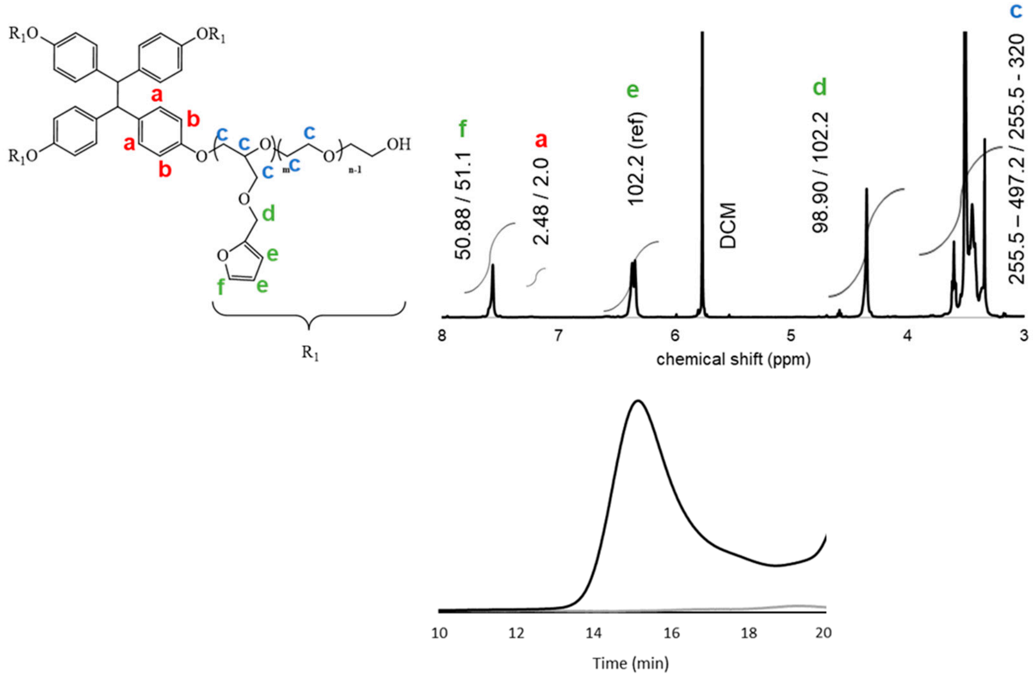

Figure S11. ^{1}H-NMR spectrum (Solvent; DMSO-d6) and GPC curve of TTP-PFG50-PEG150

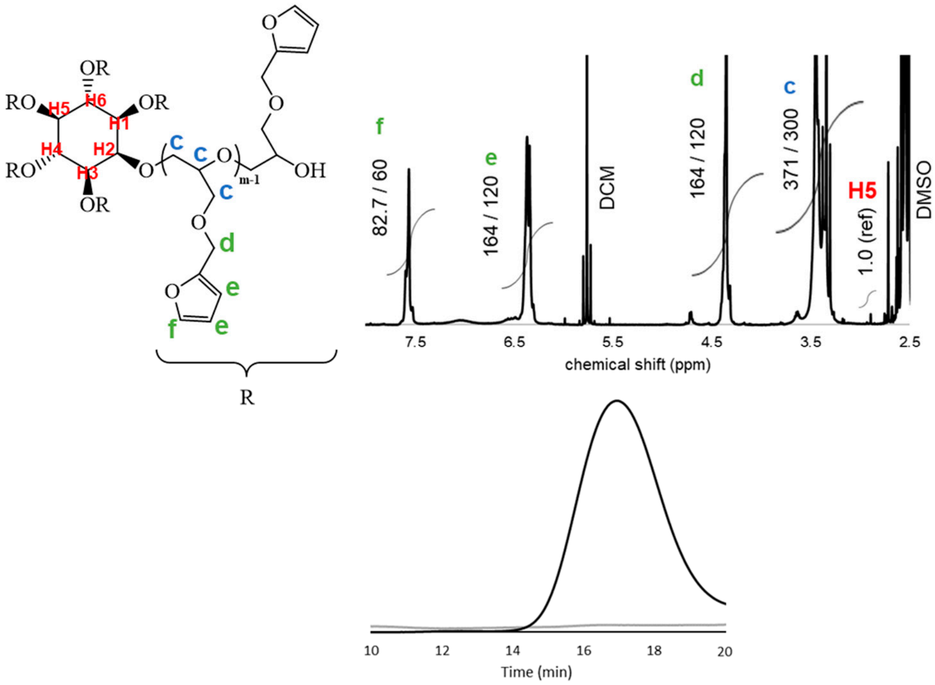

Figure S12. ^{1}H-NMR spectrum (Solvent; DMSO-d6) and GPC curve of INO-PFG10

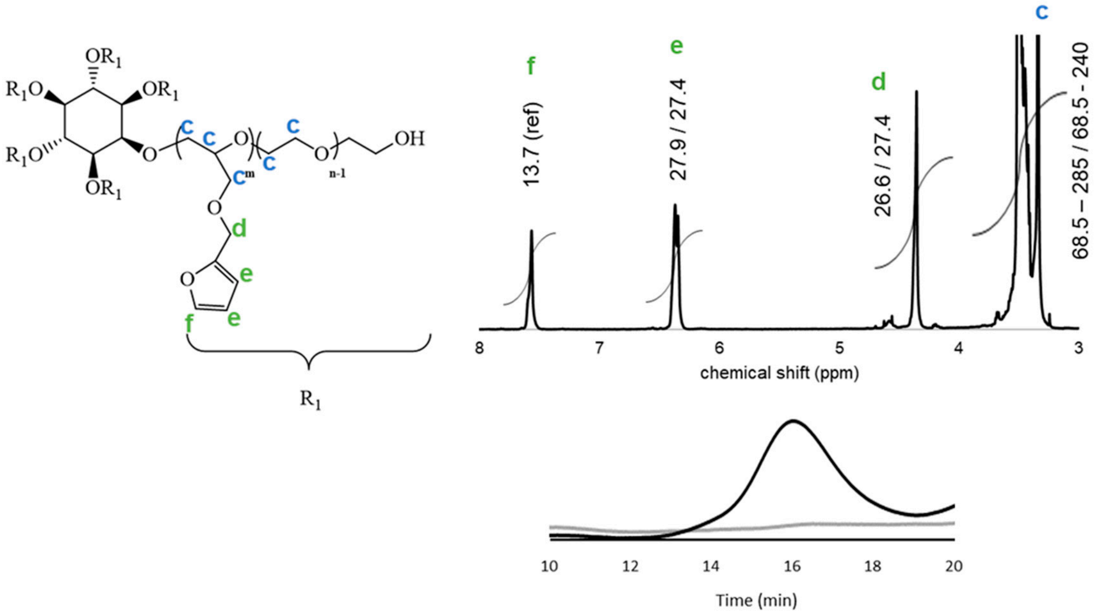

Figure S13. ^{1}H-NMR spectrum (Solvent; DMSO-d6) and GPC curve of INO-PFG10-PEG60

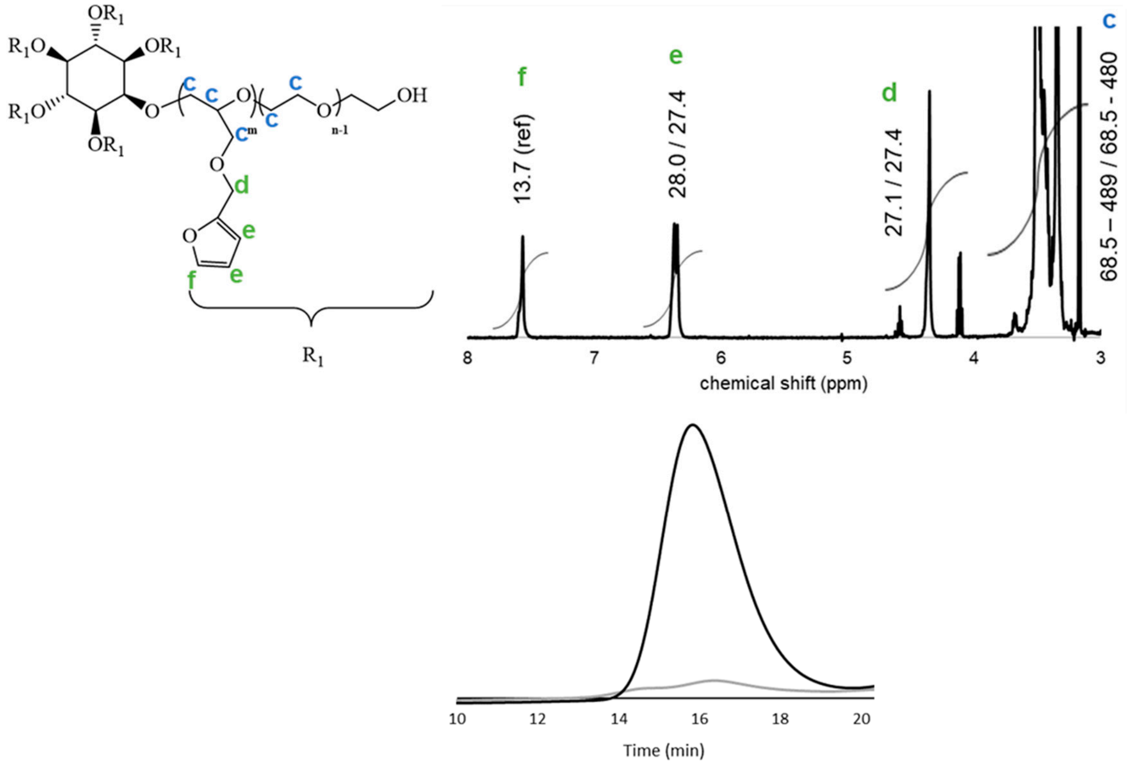

Figure S14. ^{1}H-NMR spectrum (Solvent; DMSO-d6) and GPC curve of INO-PFG10-PEG120
